# Multiparametric Magnetic Resonance Imaging Without Artificial Erection for Preoperative Staging of Primary Penile Carcinoma

**DOI:** 10.1016/j.euros.2026.06.002

**Published:** 2026-07-09

**Authors:** Marta D. Switlyk, Andreas Hopland, Andreas Tulipan, Shivanthe Sivanesan, Viktor Berge, Bjørn Brennhovd, Sigrun Dahl, Kjetil Berner, Ulrika Axcrona, Knut H. Hole

**Affiliations:** a Department of Radiology, The Norwegian Radium Hospital, Oslo University Hospital, Oslo, Norway; b Department of Urology, The Norwegian Radium Hospital, Oslo University Hospital, Oslo, Norway; c Department of Nuclear Medicine, The Norwegian Radium Hospital, Oslo University Hospital, Oslo, Norway; d Institute of Clinical Medicine (KlinMED), Faculty of Medicine, University of Oslo, Oslo, Norway; e Department of Oncology, The Norwegian Radium Hospital, Oslo University Hospital, Oslo, Norway; f Department of Pathology, The Norwegian Radium Hospital, Oslo University Hospital, Oslo, Norway

**Keywords:** Multiparametric MRI, Penile cancer, Surgery

## Abstract

**Background and objective:**

Surgery is the most common treatment option for penile carcinoma. The aim of this prospective study was to strengthen the evidence for the use of multiparametric magnetic resonance imaging (mpMRI) without artificial erection in the preoperative assessment of penile cancer.

**Methods:**

Seventy-one patients with penile cancer referred between August 2022 and September 2025 were included. Preoperative mpMRI and fluorodeoxyglucose positron emission tomography with computed tomography (FDG-PET/CT) were performed. Correlation between mpMRI and histopathology for tumor diameter and infiltration depth, along with the sensitivity and specificity of mpMRI, physical examination and FDG-PET/CT for T and N staging, were recorded. Imaging differences between human papillomavirus (HPV)-positive and HPV-negative tumors were also recorded.

**Key findings and limitations:**

mpMRI was highly sensitive and specific for the assessment of T stage, involvement of anatomical structures, and detection of skip lesions. mpMRI performed better than physical examination for T staging and had lower sensitivity but higher specificity than FDG-PET/CT for regional N staging. Moreover, significant differences between HPV-positive and HPV-negative tumors were demonstrated on functional MRI. The impact of observer variability and the need for expertise and specialized training in interpreting penile MRI are not known and may be recognized as a limitation.

**Conclusions and clinical implications:**

In this prospective single-center cohort, mpMRI without artificial erection was associated with improved preoperative locoregional assessment compared with physical examination.

## Take Home Message

Penile cancer is an uncommon malignancy. Accurate preoperative tumor assessment is essential for effective surgery planning. Clinical examination can be imprecise; therefore, advanced imaging modalities such as multiparametric magnetic resonance imaging are valuable in determining the most appropriate treatment approach.

ADVANCING PRACTICE **What does this study add?**

A physical examination alone may be inaccurate when choosing the correct surgical approach for penile cancer. Nonerectile, multiparametric MRI provides precise information about the locoregional extent of penile cancer. If incorporated as a part of the routine diagnostic workup for primary penile carcinoma, this approach will likely improve the accuracy of penile cancer surgery without compromising oncological outcomes. Minimum technical requirements should also be established. Neither MRI nor FDG-PET/CT was optimal for assessing normal-sized lymph nodes.

**Clinical Relevance**

This manuscript provides prospective evidence that modern multiparametric MRI without artificial erection may improve preoperative local staging and surgical planning in penile cancer. MpMRI demonstrated strong correlation with histopathology, accurately identified corporal and urethral invasion as well as skip lesions, and altered planned surgical management in more than one-third of patients, supporting its potential role in organ-preserving treatment strategies and personalized surgical decision-making. Associate Editor: M. Carmen Mir, M.D; PhD.

**Patient Summary**

Penile cancer is an uncommon malignancy. Evaluating the extent of the tumor through clinical examination can be difficult, and multiparametric MRI can help in selecting the best treatment strategy.

## 1 Introduction

Curative surgery is the most common treatment option for penile carcinoma, ranging from organ-sparing surgery (OSS) to more invasive procedures such as partial or total penectomy, depending on the extent of the disease [1], [2], [3]. A comprehensive assessment of tumor extent, infiltration depth, the presence of skip lesions and nodal metastases is crucial for selecting the most appropriate OSS without compromising oncological outcomes. Multiparametric magnetic resonance imaging (mpMRI) is extensively used in oncology and has experienced enormous technical progress in recent years, including the development of functional sequences and the application of artificial intelligence. The current staging of penile cancer is still largely based on physical examination, and the use of MRI for staging is limited, with most evidence coming from old, retrospective, small studies that differ technically from the modern MRI protocols in use today [3]. Primary penile cancer is a rare malignancy, and establishing minimum technical requirements for penile MRI would benefit patients and facilitate future multicenter and prospective trials by enabling more effective comparisons of outcomes. Clinical evaluation of the T stage may be challenging, and we hypothesized that mpMRI, given its well-established role in oncology, could improve staging and treatment planning for penile cancer. Therefore, the objective of this prospective study was to compare the tumor and lymph node stages on mpMRI with the results of physical examination and fluorodeoxyglucose positron emission tomography with computed tomography (FDG-PET/CT), with histology as a reference standard.

## 2 Materials and methods

### 2.1 Clinical protocol

**Fig. 1:**
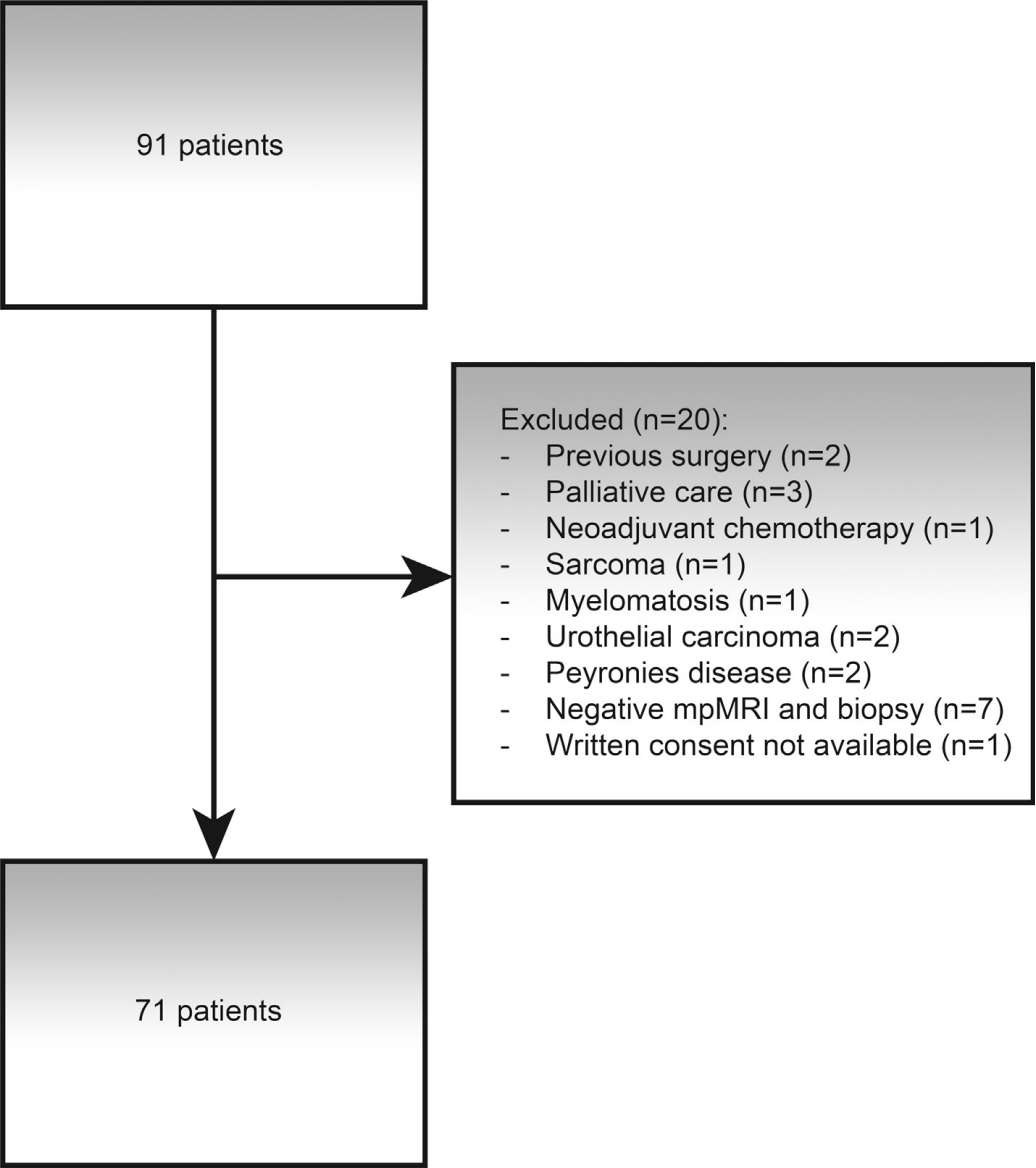
The breakdown of the patient cohort and reasons for noncompliance.

This prospective study was approved by the Regional Committee for Medical Research Ethics in South-Eastern Norway and the institutional review board (ClinicalTrials.gov identifier NCT05447273). Written informed consent was obtained from all patients. The study included 91 patients with penile squamous cell carcinoma referred to our penile cancer clinic between August 2022 and September 2025. The breakdown of the patient cohort is presented in Fig. 1. Seventy-one patients were included in the final analysis. The primary outcome was to compare T stages on mpMRI and clinical examination with those on histology, determine how preoperative mpMRI could impact intended surgical plans, and evaluate N stages on mpMRI and FDG-PET/CT with histology as the reference standard. The secondary outcome was to assess the relationship between human papillomavirus (HPV) status and imaging.

First, all patients were examined and biopsied by dedicated urologists with longstanding experience in penile cancer diagnosis and management (A.H., S.S.). Based on the clinical findings, a predefined surgical treatment plan was recorded in the medical records using a standardized form. Afterward, the patients were referred for imaging. Preoperative, non-erectile mpMRI was performed in 71 patients and FDG-PET/CT was performed in 70 patients. Imaging findings were reviewed at a multidisciplinary team conference involving urologists and radiologists, and the intended surgical treatment plan was discussed and adjusted if necessary. Finally, the surgery was performed within 2 wk following imaging. Surgical approaches included radical circumcision, partial glans resection, total glans resurfacing, glansectomy, partial, and total penectomy. The histopathology specimens were analyzed according to standardized protocols based on the American Joint Committee on Cancer format [4]. The largest tumor diameter and tumor infiltration depth/thickness were measured, and the tumors were classified into subtypes and staged according to the tumor-node-metastasis (TNM) classification [5]. Resection margins, whether positive or negative, were documented for each patient. The HPV status and HPV subtyping were obtained for all patients. HPV-DNA evaluation was performed using the Allplex HPV28 Detection Kit (Seegene Inc., Seoul, South Korea) which identifies 12 high-risk genotypes (16, 18, 31, 33, 35, 39, 45, 51, 52, 56, 58, 59), and types 66 and 68, along with 14 additional HPV types (26, 53, 69, 73, 82, 6, 11, 40, 42, 43, 44, 54, 61, 70). The management of lymph nodes included surveillance, dynamic sentinel node biopsy (DSNB), inguinal lymph node dissection (ILND), and pelvic lymph node dissection.

Lymph node surgery was not performed in nine patients, based on negative clinical examination and negative imaging results. Therefore, the histological N stage was not available for these patients, and they were excluded from the respective analyses. Moreover, histopathological assessments of tumor thickness/infiltration depth, largest tumor diameter, and ingrowth in the urethra were not available for eight, nine, and two patients, respectively, in our study.

### 2.2 Analysis of mpMRI and FDG-PET/CT

The complete MRI protocol is described in Supplementary Tables 1–3. mpMRI was performed using a 3-T magnet (MAGNETOM Vida Fit, Siemens Healthineers, Erlangen, Germany). In a few patients with metallic implants, examinations were conducted using a 1.5-T magnet (MAGNETOM Sola, Siemens Healthineers, Erlangen, Germany) following a similar protocol. Examination was performed without artificial erection, bowel relaxants, or rectal emptying. The penis was positioned in a normal anatomical location, pointing downward along the midline. The prepuce was in a normal position and pulled down over the glans. The MR images were prospectively reviewed by one of two uroradiologists (MDS, KHH) with 17 and 28 yr of experience. The T and N stages, according to the TNM classification [5] and the largest diameter and thickness/infiltration depth of the tumor were determined. The regions of interest within the tumor were drawn on apparent diffusion coefficient (ADC) maps and dynamic contrast-enhanced MRI (DCE-MRI) perfusion. The DCE-MRI data were analyzed by conventional classification of the kinetic curves (types 1–3). The radiologist performed a comprehensive, experience-based evaluation of N stage on MRI, and the lymph node was considered metastatic if one or more of the following criteria, often in combination, were met: a short axis diameter >15 mm, a round shape without a fatty hilum, and evidence of intralesional necrosis or extranodal extension.

FDG-PET/CT examinations were performed using a GE Discovery MI PET/CT (GE Healthcare, Waukesha, WI, USA) with a four-ring digital PET detector. The complete PET/CT protocol is described in Supplementary Table 4. FDG-PET/CT images were prospectively reviewed by a nuclear medicine physician (AT) with 18 yr of experience. The standardized uptake values of the primary tumor and N and M stages were recorded. The nuclear medicine physician performed a comprehensive, experience-based evaluation of N stage on FDG-PET/CT, and the lymph node was considered metastatic if one or more of the following criteria, often in combination, were met: high uptake above the liver background, a round shape without a fatty hilum and uptake distinctly above background uptake in other regional lymph nodes, and a short axis diameter greater than 15 mm.

### 2.3 Statistical analyses

The data were analyzed using STATA (version 18, StataCorp, TX, USA) and SPSS (version 30; IBM Corp., Armonk, NY, USA). The Spearman rank-order correlation was applied to continuous data. The Mann-Whitney U test or independent samples *t* test was performed to assess the associations between continuous variables and categorical data. The Pearson chi-square test was applied for categorical variables, and comparisons among imaging, physical examination, and histopathological features were conducted in a descriptive manner. Cohen’s kappa (κ) for nominal variables and quadratic weighted κ for ordinal variables were estimated to evaluate the concordance between the imaging, physical examination, and histopathological features. The interpretations of the correlation coefficients (ρ), effect sizes (r) and κ values are presented in Supplementary Table 5 [6], [7], [8]. The sensitivity, specificity, positive predictive value (PPV), negative predictive value (NPV), and 95% confidence intervals (CIs) were calculated, with inconclusive results incorporated into the analyses [9], [10]. 95% CIs were calculated using logit transformation, and the Clopper-Pearson method was used for extreme values (close to 0 or 1). Statistical tests were two-sided with a nominal significance level of 0.05, with no adjustments for multiplicity applied.

## 3 Results

### 3.1 Study population

The details are listed in Table 1, including high-risk HPV genotypes. All the HPV genotypes are presented in Supplementary Table 6.

**Table 1:** Study characteristics

**Variables**	**Patients (n** = **71)**
Age (years)	72 (58-78)
	
HPV-negative	30 (42)
HPV-positive	41 (58)
16	28 (68)^a^
18	3 (7)^a^
31	5 (12)^a^
33	1 (2)^a^
39	2 (5)^a^
45	2 (5)^a^
52	1 (2)^a^
56	1 (2)^a^

Treatment^b^	
Circumcision	8 (11)
Partial glans resection	3 (4)
Total glans resurfacing	3 (4)
Glansectomy	21 (30)
Partial penectomy	29 (41)
Total penectomy	8 (11)

Histopathology	
Thickness/infiltration depth (mm)^c^	9 (5-13)
Largest diameter (mm)^d^	29 (22-40)
PeIN only	5 (7)
Squamous cell carcinoma subtypes	
HPV-associated	25 (35)
Basaloid	10 (14)
Clear cell	1 (1)
Warty-basaloid	1 (1)
Sarcomatoid	1 (1)
HPV-independent/usual type	28 (39)

MpMRI	
Thickness/infiltration depth (mm)	10 (6-14)
Largest diameter (mm)	27 (20-38)
ADC in all tumors (µmm^2^/s)	873 (796-999)
ADC in HPV-positive tumors (µmm^2^/s)	831 (743-889)
ADC in HPV-negative tumors (µmm^2^/s)	996 (882.5-1079)
Skip lesions	3 (4)

FDG-PET/CT^e^	
SUV in all tumors	14.6 (10.1-21.1)
SUV in HPV-positive tumors	14.9 (10-20.4)
SUV in HPV-negative tumors	14.7 (9.4-22.4)

Co-infection with multiple HPV subtypes was common. Continuous variables are described by median (interquartile range); all other results are number (percentage) of patients. The percentages may not add up to 100 because of rounding.

ADC = apparent correlation coefficient; FDG-PET/CT = fluorodeoxyglucose positron emission tomography with computed tomography; HPV = human papillomavirus; mpMRI = multiparametric magnetic resonance imaging; PeIN = penile intraepithelial neoplasia; SUV = standardized uptake value.

a Percentages of patients with HPV-positive disease (*n* = 41).

b Some patients might undergo more than one treatment approach.

c Missing histopathological data (*n* = 8).

d Missing histopathological data (*n* = 9).

e FDG-PET/CT data not available (*n* = 1).

### 3.2 Infiltration depth and tumor diameter

**Fig. 2:**
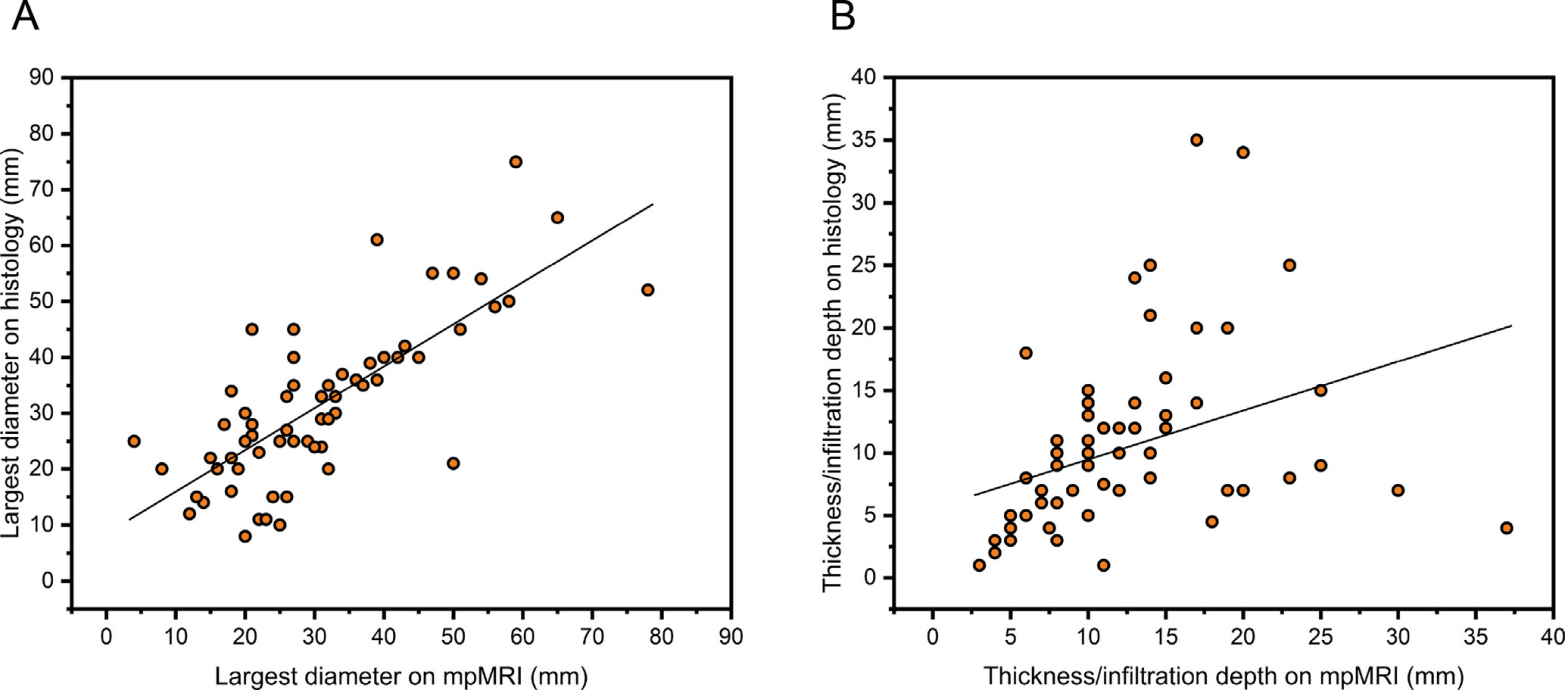
**Correlation between multiparametric magnetic resonance imaging (mpMRI) findings and histopathology for the largest tumor diameter (A) and infiltration depth (B). Scatterplots show a strong correlation between mpMRI and histology for the largest tumor diameter (ρ =0.74, *p*< 0.001) and a moderate correlation for infiltration depth (ρ = 0.55, *p*** **< 0.001)**. mpMRI = multiparametric magnetic resonance imaging.

The correlations between mpMRI and histology for the infiltration depth and largest tumor diameter are presented in Fig. 2. There was a strong correlation between mpMRI and histology for the largest tumor diameter (ρ = 0.74, *p <* 0.001) and a moderate correlation for infiltration depth (ρ = 0.55, *p <* 0.001).

### 3.3 T staging

The findings are presented in Table 2. Good agreement between mpMRI and histopathology was observed for the involvement of the corpus spongiosum (κ = 0.72, *p <* 0.001). The sensitivity and specificity of mpMRI for the involvement of the corpus spongiosum were 96% and 73%, respectively. Good agreement was found for the involvement of the tunica albuginea/corpus cavernosum (κ = 0.80, *p <* 0.001), with a sensitivity of 100% and specificity of 93%. Good agreement was observed for the involvement of the penile urethra (κ = 0.71, *p <* 0.001), with a sensitivity and specificity of 79% and 91%, respectively.

**Table 2:** Association between findings on multiparametric magnetic resonance imaging (mpMRI), physical examination, fluorodeoxyglucose positron emission tomography with computed tomography (FDG-PET/CT) and histopathology

MpMRI	Histopathology				κ	Sensitivity (95% CI)	Specificity (95% CI)	PPV (95% CI)	NPV (95% CI)
Involvement of corpus spongiosum	Yes		No		0.72	96 (84–99)	73 (53–87)	88 (75–94)	90 (68–98)
Yes	43 (61)		6 (8)						
No	2 (3)		19 (27)						
Inconclusive	0 (0)		1 (1)						
Involvement of urethra^a^	Yes		No		0.71	79 (58–91)	91 (78–97)	86 (65–96)	89 (76–95)
Yes	19 (27)		3 (4)						
No	5 (7)		41 (58)						
Inconclusive	0 (0)		1 (1)						
Involvement of corpus cavernosum	Yes		No		0.80	100 (69–100)	93 (84–98)	71 (43–89)	100 (94–100)
Yes	10 (14)		4 (6)						
No	0 (0)		57 (80)						
T stage	≤ T1	T2		T3	0.73^b^				
≤ T1	20 (28)	2 (3)		0 (0)		77 (57–89)	96 (84–99)	91 (69–98)	90 (77–96)
T2	5 (7)	29 (41)		0 (0)		83 (66–92)	83 (67–92)	85 (69–94)	83 (67–92)
T3	0 (0)	4 (6)		10 (14)		100 (69–100)	92 (82–97)	71 (43–89)	100 (94–100)
Inconclusive	1 (1)	0 (0)		0 (0)					
Lymph node metastases^c^	Yes		No		0.53	67 (46–83)	82 (66–91)	80 (57–92)	84 (68–93)
Yes	16 (23)		4 (6)						
No	6 (8)		31 (44)						
Inconclusive	2 (3)		3 (4)						
N stage^c^	N0	N1	N2	N3	0.36^b^				
N0	31 (44)	5 (7)	0 (0)	1 (1)		79 (64–90)	65 (44–82)	84 (68–93)	75 (52–89)
N1	3 (4)	6 (8)	1 (1)	2 (3)		46 (22–72)	82 (68–90)	50 (24–76)	89 (77–95)
N2	1 (1)	0 (0)	2 (3)	2 (3)		67 (15–96)	86 (75–93)	40 (10–81)	98 (87–100)
N3	0 (0)	1 (1)	0 (0)	2 (3)		29 (7–68)	89 (77–95)	67 (15–96)	91 (79–96)
Inconclusive	3 (4)	2 (3)	0 (0)	0 (0)					

**Physical examination**	**Histopathology**				**κ**	**Sensitivity** **(95% CI)**	**Specificity (95% CI)**	**PPV** **(95% CI)**	**NPV** **(95% CI)**

T stage	≤ T1	T2		T3	0.56^b^				
≤T1	15 (21)	5 (7)		0 (0)		58 (38–75)	89 (76–95)	75 (52–89)	78 (65–88)
T2	10 (14)	19 (27)		3 (4)		54 (38–70)	64 (47–78)	59 (42–75)	59 (43–73)
T3	1 (1)	11 (15)		7 (10)		70 (37–90)	80 (68–89)	37 (18–60)	94 (83–98)

**FDG–PET/CT**	**Histopathology**				**κ**	**Sensitivity** **(95% CI)**	**Specificity** **(95% CI)**	**PPV** **(95% CI)**	**NPV** **(95% CI)**

Lymph node metastases^c^, ^d^	Yes		No		0.33	75 (54–89)	55 (39–70)	60 (42–76)	81 (61–92)
Yes	18 (25)		12 (17)						
No	5 (7)		21 (30)						
Inconclusive	1 (1)		5 (7)						
N stage^c^, ^d^	N0	N1	N2	N3	0.13^b^				
N0	21 (30)	4 (6)	0 (0)	1 (1)		54 (38–69)	78 (57–91)	81 (61–92)	50 (34–66)
N1	11 (15)	5 (7)	2 (3)	4 (6)		38 (17–66)	65 (51–77)	23 (10–45)	80 (64–90)
N2	1 (1)	3 (4)	1 (1)	1 (1)		33 (4–85)	92 (81–96)	17 (2–64)	96 (86–99)
N3	0 (0)	1 (1)	0 (0)	1 (1)		14 (2–59)	98 (88–100)	50 (6–94)	90 (79–96)
Inconclusive	5 (7)	1 (1)	0 (0)	0 (0)					

CI = confidence interval; FDG-PET/CT = fluorodeoxyglucose positron emission tomography with computed tomography; κ = kappa**;** mpMRI = multiparametric magnetic resonance imaging; NPV = negative predictive value; PPV = positive predictive value.

a Missing histopathological data (*n* = 2).

b Quadratic-weighted κ was estimated for ordinal variables; all other κ values are unweighted. Categorical variables are described by the number (percentage) of patients.

c Missing histopathological data (*n* = 9).

d FDG-PET/CT data not available (*n* = 1).

Good agreement and a statistically significant association between mpMRI and histopathology were found for the overall T stage (κ = 0.73, *p <* 0.001). The sensitivity and specificity for the diagnosis of T1, T2 and T3 disease were 77% and 96%, 83% and 83%, and 100% and 92%, respectively.

Compared with mpMRI, physical examination showed moderate agreement for the overall T stage (κ = 0.56, *p <* 0.001) and had lower sensitivity and specificity for diagnosing T1, T2 and T3 disease. Preoperative mpMRI led to changes in planned surgical procedures in 25 of 71 patients (35%; Table 3). Except for two patients, all patients in our study (*n* = 69) had free resection margins. All patients whose surgical procedures were changed after MRI, whether to a more organ-preserving surgery or a less organ-sparing approach, had free resection margins.

**Table 3:** Summary of 25 cases in which preoperative multiparametric magnetic resonance imaging (mpMRI) findings resulted in changes in surgical management

**Planned surgery**	**Changes in surgical management after mpMRI**	**Number of patients**
Total glans resurfacing	↓ Partial glans resection^a^	1
Glansectomy	↓ Partial glans resection^a^	1
Partial penectomy	↓ Partial glans resection or glansectomy^a^	5
Partial penectomy	↓ Partial penectomy with corporal length sparing^a^	1
Total penectomy	↓ Partial penectomy^a^	1
		
Circumcision	↑ Circumcision and partial glans resection or glansectomy^b^	2
Circumcision and partial glans resection	↑ Partial penectomy^b^	1
Partial glans resection	↑ Glansectomy or partial penectomy^b^	2
Total glans resurfacing	↑ Glansectomy^b^	2
Glansectomy	↑ Partial or total penectomy^b^	9

mpMRI = multiparametric magnetic resonance imaging.

a Decreased extent of penile surgery

b Increased extent of penile surgery.

### 3.4 Skip lesions

**Fig. 3:**
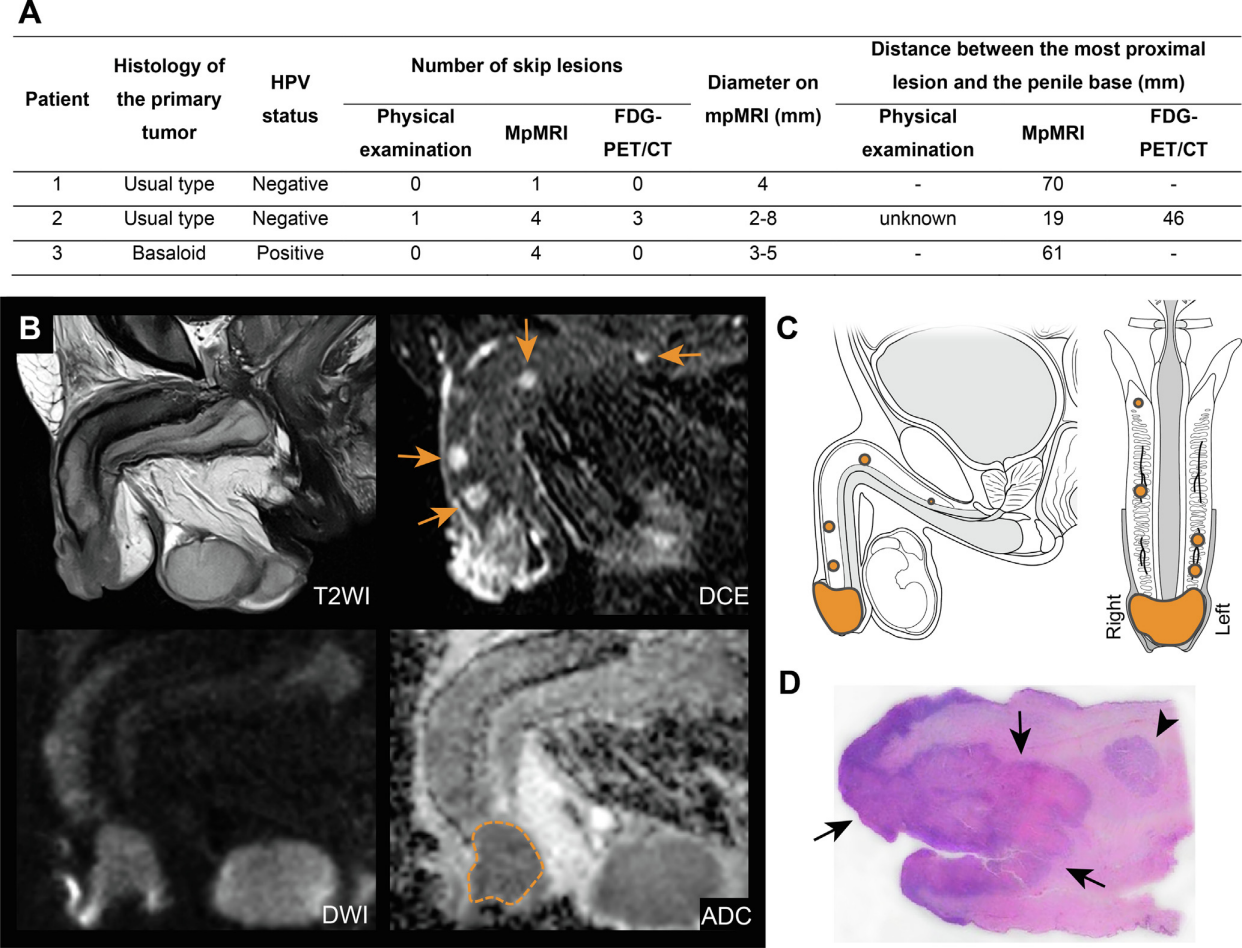
**Characteristics of three patients with penile skip lesions (A). An example of the detection of skip lesions on multiparametric magnetic resonance imaging (mpMRI) (B). A large T3 tumor involving the glans, urethra, and corpus cavernosum is shown on T2-weighted imaging (T2WI), dynamic contrast-enhanced (DCE) perfusion, diffusion-weighted imaging (DWI), and apparent diffusion coefficient (ADC) (B, staple line). The human papillomavirus (HPV) status was negative, and the tumor demonstrated relatively high diffusion (ADC = 1164 mmm^2^/s). There are four skip lesions in the corpora cavernosa, two on each side, most clearly demonstrated on DCE perfusion (B, arrows). The location and number of skip lesions are crucial for planning surgery and preventing tumor recurrence. These tiny lesions were impossible to detect on physical examination, and the tumor was clinically staged as T2. Schematic drawing displaying the location of the primary tumor and the skiplesions presented under the multidisciplinary meeting for the urologists (C). The drawing was available in the picture archiving and communication system (PACS) along with the mpMR images, allowing precise planning of the surgery. Total penectomy was performed. A large T3 tumor (arrows) and a skip lesion (arrowhead) are displayed on the photomicrograph of a whole-mount hematoxylin and eosin-stained section (D), corresponding to the mpMRI findings.** ADC = apparent diffusion coefficient; DCE = dynamic contrast-enhanced; DWI = diffusion-weighted imaging; HPV = human papillomavirus; mpMRI = multiparametric magnetic resonance imaging; PACS = picture archiving and communication system; T2WI = T2-weighted imaging.

Skip lesions in the corpora cavernosa were identified in three patients (Fig. 3). Skip lesions were significantly associated with the involvement of the corpus cavernosum by primary tumor (*p* = 0.004), T stage (*p* = 0.014), N stage (*p* = 0.013) and the presence of nodal metastases in general (*p* = 0.025; Supplementary Table 7).

### 3.5 N staging

The findings are presented in Table 2. Moderate agreement and a statistically significant association between mpMRI and histopathology were found for the overall detection of nodal metastases (κ = 0.53, *p <* 0.001). The sensitivity and specificity of mpMRI for the detection of nodal metastases were 67% and 82%, respectively.

Fair agreement and a statistically significant association between FDG-PET/CT and histopathology were found for the detection of lymph node metastases (κ = 0.33, *p* = 0.004). Compared with MRI, FDG-PET/CT had higher sensitivity and lower specificity for the detection of nodal metastases (75% and 55%, respectively).

Except for two patients, all false-negative and false-positive results on mpMRI and FDG-PET/CT occurred in normal-sized inguinal lymph nodes (<15 mm in the short axis on imaging). Of 62 patients who underwent lymph node surgery, mpMRI missed nodal metastases in six cases (10%) and FDG-PET/CT in five cases (8%).

### 3.6 Reporting of inconclusive results

We incorporated the inconclusive results into the analyses following the equation proposed by Garcia-Romero et al [9], as this approach likely provided the most realistic accuracy statistics. The sensitivity, specificity, PPV and NPV calculated in alternative analyses where inconclusive results were either excluded or grouped with positive or negative findings are presented in Supplementary Tables 8–10.

### 3.7 Imaging findings and HPV status

The findings are illustrated in Supplementary Fig. 1. ADC values were significantly lower in HPV-positive tumors than in HPV-negative tumors (r = 0.5, t = 4.311, *p <* 0.001; Supplementary Table 11). No other findings were associated with HPV status (Supplementary Tables 12–14).

## 4 Discussion

This prospective study revealed a significant association between non-erectile mpMRI and histopathology in patients with penile carcinoma. mpMRI accurately assessed tumor size and infiltration depth and performed better than clinical examination for evaluating T stage. Significant associations between imaging and histopathology were found for N stage. Compared with FDG-PET/CT, mpMRI had lower sensitivity but higher specificity for the detection of nodal metastases. In addition, significant differences between HPV-positive and HPV-negative tumors were detected on mpMRI.

According to the European Association of Urology–American Society of Clinical Oncology guidelines for the diagnosis and staging of penile cancer, MRI can be helpful when the OSS is planned or when there is a suspicion of corpus cavernosum invasion [3], [11]. According to the guidelines, physical examination is a reliable method for estimating tumor size and stage, and MRI does not outperform physical examination, at least in the ability to differentiate between T1 and T2 disease [3]. This recommendation is based on the study conducted by Lont et al more than 20 yr ago, with a limited number of patients and principal technical differences from the MRI protocols currently used [12]. This study concluded that tumor size is determined with the highest precision by physical examination and that MRI is the least precise, being outperformed both by the physical examination and ultrasound [12]. However, in our study, mpMRI demonstrated superior performance to physical examination in assessing T stage owing to recent advancements in MRI technique, improved image quality, and the ability to detect and define tumor. Physical examination was particularly challenging for ulcerative and painful lesions, obese patients, superimposed infection, tumor invasion into the penile urethra and identifying skip lesions. In our study, preoperative mpMRI led to changes in planned surgical procedures in 35% (25/71) of the patients.

Skip lesions in the corpora cavernosa were present in 4% of the patients, and all the lesions were precisely identified by mpMRI (Fig. 3). Detecting skip lesions is crucial for surgical planning, as they determine the proximal resection margins and may convert an OSS to partial or total penectomy. However, in our study, most of them could not be identified by physical examination or FDG-PET/CT, making MRI the only reliable method for detection. In agreement with prior studies, skip lesions were identified in patients with advanced tumors and more aggressive disease [13]. From a surgical perspective, the detection of skip lesions may prevent inadequate resections and reduce the need for reoperation or subsequent oncological treatment.

A systematic review and meta-analysis by Krishna et al. [14] evaluated data from eight studies to assess the accuracy of MRI for penile cancer staging [12], [15], [16], [17], [18], [19], [20], [21]. However, many of these studies had retrospective or unclear designs, and the number of patients was as low as six, making interpretation of the outcome difficult. Compared with the two largest prospective studies [15], [18], mpMRI in our study had higher sensitivity and higher or similar specificity for the detection of T3 disease (100% and 93% in our study vs 74–82% and 79–99% in previous studies, respectively) and higher sensitivity and specificity for the involvement of the urethra (79% and 91% in our study vs 63% and 87% in previous studies, respectively). In both these studies, MRI was performed with artificial erection.

Our results revealed a significant correlation between mpMRI and histopathology for tumor size and infiltration depth. The largest tumor diameter and thickness/infiltration depth varied between 8 and 75 mm and between 1 and 35 mm, respectively. Thus, mpMRI reliably assessed tumor size and proximal tumor extent in both small superficial lesions and large advanced tumors. Some difficulties in measuring lesions in a standardized way on MRI and histopathology have been experienced, possibly explaining the lower agreement for infiltration depth.

There was a significant association and fair/moderate agreement between mpMRI and histopathology for N stage and the overall ability to detect regional lymph node metastases. Few data are available on the diagnostic accuracy of MRI for the detection of nodal metastases in penile cancer [17], [22], [23]. A study by Lucchesi et al reported that MRI correctly staged 87% of patients; however, they included only 15 cases [17]. According to our results, mpMRI correctly staged 66% of patients with available histopathological data and had a sensitivity of 67% and a specificity of 82% for the overall detection of nodal metastases. In comparison, FDG-PET/CT showed fair agreement for the overall detection of nodal metastases, along with higher sensitivity but lower specificity than mpMRI. This result can be explained by the somewhat limited ability of FDG-PET/CT to differentiate reactive lymph nodes from metastatic lymph nodes and the limited ability of MRI to detect small, focal metastases [24]. Among the 62 patients who underwent lymph node surgery, mpMRI missed nodal metastases in 10% (6/62), while FDG-PET/CT in 8% (5/62). In our study, except for two patients, all false-negative and false-positive results occurred in normal-sized inguinal lymph nodes. These findings emphasize the importance of DSNB in radiologically negative lymph nodes. In contrast, MRI may help determine which patients should undergo DSNB and which should undergo ILND. A recently published meta-analysis reported higher sensitivity and specificity of FDG-PET/CT than did our report; however, most studies had retrospective designs, and there was considerable heterogeneity among the studies [25]. In our study, the specificity of FDG-PET/CT increased when inconclusive results were classified along with negative findings, indicating that many uncertain lymph nodes were in fact reactive (Supplementary Table 9). Nevertheless, FDG-PET/CT remains an important modality for the evaluation of patients with widespread cancer and distant metastases.

In recent years, an increasing incidence of penile cancer has been noted due to an increase in HPV-related tumors [3]. The percentage of HPV-positive disease in our study was higher than that in previously published studies (58% in our study vs 30–50% in previous studies) [26], [27], [28]. We found significantly higher ADC in HPV-negative tumors, and our results are in line with findings on head and neck cancer [29]. The relationship between functional MRI and HPV expression patterns could be useful for selecting patients with poorer prognosis and higher recurrence risk, but more evidence is needed [11].

Overall, non-erectile mpMRI was well tolerated by all patients, regardless of age or health status. No patients were excluded because of suboptimal image quality. Functional sequences are frequently robust to motion artifacts and were particularly useful when scanning patients unable to lie motionless during an MRI scan [11]. Patients with strong claustrophobia may find it difficult to complete the entire examination, but using wide-bore MRI systems, good patient information and sedation when needed can address this issue. The cost-effectiveness of penile MRI is difficult to assess due to the scarce data available. However, the incidence of penile cancer is low, and there are potential benefits arising from MRI-based assessment, such as staging and delineation of tumor extent [11].

This study has several strengths and limitations. The strengths of this study include its prospective design, high technical image quality, and thorough histological correlation. In contrast, the impact of observer variability and the need for education, specialized training, and expertise in interpreting and reporting penile MRI is not known and needs to be corroborated in future studies [11]. Moreover, the histological N stage was available only for patients who underwent lymph node surgery, and patients who did not undergo such surgery (*n* = 9) were excluded based on negative clinical examination and negative imaging results. Consequently, estimates of N staging accuracy may be affected by verification/selection bias and may not be generalizable to all patients undergoing imaging. Finally, the radiologists did not directly compare the mpMRI and FDG-PET/CT scans; however, these scans were stored in the picture archiving and communication system, and some bias cannot be excluded.

## 5 Conclusion

In evaluating the T stage, mpMRI was associated with improved preoperative assessment compared with physical examination and led to changes in surgical procedures in more than one-third of patients. Neither mpMRI nor FDG-PET/CT provided optimal assessment of normal-sized lymph nodes.

  ***Author contributions:*** The first and corresponding authors had full access to all the data in the study and take responsibility for the integrity of the data and the accuracy of the data analysis.

  *Study concept and design:* Switlyk, Hopland, Hole.

*Acquisition of data:* All authors.

*Analysis and interpretation of data:* Switlyk, Tulipan, Hopland.

*Drafting of the manuscript:* Switlyk, Hopland.

*Critical revision of the manuscript for important intellectual content:* All authors.

*Statistical analysis:* Switlyk.

*Obtaining funding:* None.

*Administrative, technical, or material support:* None.

*Supervision:* Switlyk, Hole.

*Other:* None.

  ***Financial disclosures:*** Marta D. Switlyk certifies that all conflicts of interest, including specific financial interests and relationships and affiliations relevant to the subject matter or materials discussed in the manuscript (eg, employment/affiliation, grants or funding, consultancies, honoraria, stock ownership or options, expert testimony, royalties, or patents filed, received, or pending), are the following: None.

  ***Funding/Support and role of the sponsor:*** None.
