# Supplementary material for: Multiparametric Magnetic Resonance Imaging Without Artificial Erection for Preoperative Staging of Primary Penile Carcinoma
Source: Eur Urol Open Sci. 2026 Jul 9;90:111–20. doi: 10.1016/j.euros.2026.06.002 (PMC13377138; doi:10.1016/j.euros.2026.06.002)
Supplement: Supplementary Data 1 [file mmc1.docx]

**Supplementary Figure 1.** Apparent diffusion coefficient (ADC) on multiparametric magnetic resonance imaging (mpMRI) and histopathological findings in human papillomavirus (HPV)-positive and HPV-negative tumors. HPV-positive tumor is cell dense with homogenous, low diffusion (A). A photomicrograph shows tumor tissue arranged in solid, highly cellular sheets with scarce stromal tissue between the cells. Immunochemistry revealed a positive reaction for p16 (B). The diffusion in HPV-negative tumor is higher (A), and the photomicrograph shows tumor tissue with prominent stroma and cells arranged in round nests. Immunochemistry revealed no reaction for p16 (B). Box plots with data points show that ADC values are significantly lower in HPV-positive tumors than in HPV-negative tumors (r=0.5, t=4.311, p<0.001) (C).

**
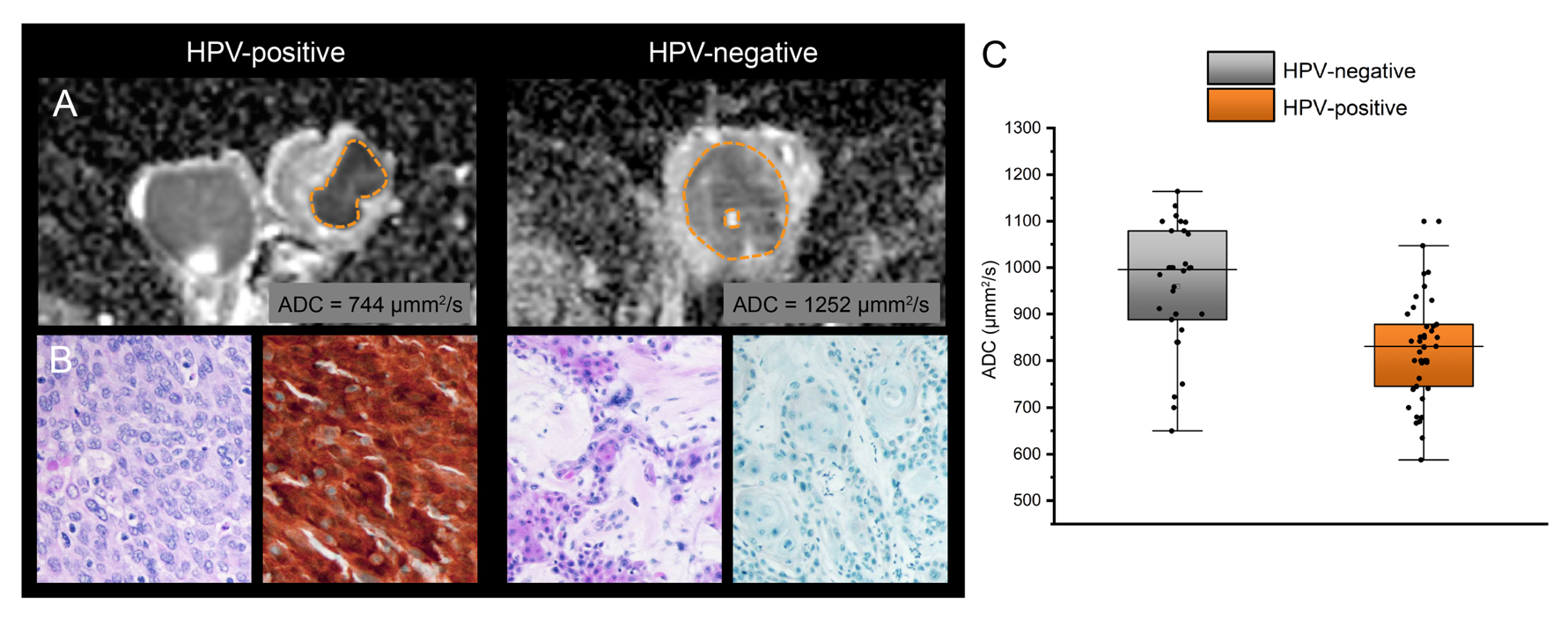
**

**Supplementary Table 1.** Multiparametric magnetic resonance imaging (mpMRI) protocol for the assessment of primary penile carcinoma – general information.

| - MpMRI was performed using a 3-T magnet with a 32-channel phased-array coil - The imaging protocol consisted of high-resolution morphological and functional sequences covering the penis and lower pelvis - Examination was performed without artificial erection, bowel relaxants or rectal emptying - The penis was in a normal anatomical location, pointing downward along the midline - The prepuce was in a normal position and pulled down over the glans - In a few patients with metallic implants, examinations were conducted using a 1.5-T magnet following a similar protocol. |
| --- |

mpMRI = multiparametric magnetic resonance imaging. T = tesla

**Supplementary Table 2.** Multiparametric magnetic resonance imaging (mpMRI) protocol for the assessment of primary penile carcinoma (3-T Vida Fit, Siemens) [11].

| **MpMRI parameters** | **T2W DL** | **DWI RESOLVE** | **DWI** | **DCE-MRI**  **TWIST-VIBE** | **T1W after Gd** |
| --- | --- | --- | --- | --- | --- |
| Pulse sequence | 2D SE | Small FOV multi-shot EPI | STIR-EPI | 3D spoiled GE Dixon | 3D spoiled GE Dixon |
| Coverage area | Primary tumor | Primary tumor | Primary tumor, inguinal and pelvic lymph nodes | Primary tumor | Primary tumor, inguinal and pelvic lymph nodes |
| Plane | Sag, Cor, Tra | Sag, Cor, Tra | Tra | Tra | Tra |
| Slice thickness (mm)  Acquired  Interpolated | 2.0 | 2.3 | 5.0 | 2.2  1.1 | 1.2  0.6 |
| FOV (mm) | 230 | 149 | 300 x 370 | 169 | 300 x 306 |
| TE (ms) | 99 | 55 | 64 | 1.35 and 2.58 | 2.46 and 3.69 |
| TR (ms)  TI (ms) | 2620  - | 3040  - | 7190  240 | 4.4  - | 5.83  - |
| Flip angle | 128° | 180° | - | 13° | 12° |
| Gap (mm) | 0.0 | 0.76 | 1.0 | 0.44 | 0.24 |
| NEX | 1 | b0 = 2  b800 = 4 | b0 = 3  b800 = 3 | 1 | 2 |
| In-plane resolution (mm x mm)  Acquired  Interpolated | 0.72 x 0.72  0.36 x 0.36 | 1.47 x 1.47  0.74 x 0.74 | 2.5 x 2.5  1.25 x 1.25 | 1.32 x 1.32  0.66 x 0.66 | 0.74 x 0.82  0.37 x 0.37 |
| ETL | 19 | 69 | 60 | - | - |
| Bandwidth (Hz/Px) | 422 | 980 | 2111 | 810 | 668 |
| Matrix  Acquired  Interpolated | 320 x 320  640 x 640 | 102 x 102  204 x 204 | 120 x 148  296 x 296 | 128 x 128  256 x 256 | 374 x 416  748 x 832 |
| R-factor | 4 | 2 | 2 | 4 | 4 |
| b-values | - | Acquired 0 and 800, calculated 1400 | Acquired 0 and 800 | - | - |
| Time resolution (sec) | - | - | - | 9.38 | - |
| Acquisition time (min) | 1:20 | 3:50 | 2:22 | 5:12 | 6:05 |

mpMRI = multiparametric magnetic resonance imaging. DL = deep learning. RESOLVE = readout segmentation of long variable echo-trains. TWIST = time-resolved angiography with interleaved stochastic trajectories. VIBE = volume-interpolated breath-hold examination. DWI = diffusion-weighted imaging. DCE-MRI = dynamic contrast-enhanced magnetic resonance imaging. Gd = gadolinium. SE = spin echo. FOV = field of view. EPI = echo-planar imaging. STIR = short tau inversion recovery. GE = gradient echo. Sag = sagittal. Cor = coronal. T = tesla. Tra = transversal. TE = echo time. TR = repetition time. TI = inversion time. NEX = number of excitations. ETL = echo train length. R-factor = reduction factor using parallel imaging acceleration.

**Supplementary Table 3.** Multiparametric magnetic resonance imaging **(**mpMRI) protocol for the assessment of primary penile carcinoma (1.5-T Sola, Siemens) [11].

| **MpMRI parameters** | **T2W DL** | **DWI**  **ZOOMit^PRO^** | **DWI** | **DCE-MRI**  **TWIST-VIBE** | **T1W after Gd** |
| --- | --- | --- | --- | --- | --- |
| Pulse sequence | 2D SE | Reduced FOV EPI | STIR-EPI | 3D spoiled GE Dixon | 3D spoiled GE Dixon |
| Coverage area | Primary tumor | Primary tumor | Primary tumor, inguinal and pelvic lymph nodes | Primary tumor | Primary tumor, inguinal and pelvic lymph nodes |
| Plane | Sag, Cor, Tra | Sag, Cor, Tra | Tra | Tra | Tra |
| Slice thickness (mm)  Acquired  Interpolated | 2.0 | 2.3 | 5.0 | 2.2  1.1 | 1.4  0.7 |
| FOV (mm) | 230 | 150 | 250 x 370 | 200 | 300 x 306 |
| TE (ms) | 94 | 71 | 63 | 2.39 and 4.77 | 2.39 and 4.77 |
| TR (ms)  TI (ms) | 2820  - | 2800  - | 5180  180 | 6.4  - | 5.83  - |
| Flip angle | 110° | - | - | 13° | 12° |
| Gap (mm) | 0.0 | 0.58 | 0.0 | 0.44 | 0.28 |
| NEX | 2 | b0 = 8  b800 = 16 | b0 = 3  b800 = 6 | 1 | 2 |
| In-plane resolution (mm x mm)  Acquired  Interpolated | 0.80 x 0.80  0.40 x 0.40 | 1.47 x 1.47  0.74 x 0.74 | 2.98 x 2.98  1.49 x 1.49 | 1.56 x 1.56  0.78 x 0.78 | 0.74 x 0.82  0.37 x 0.37 |
| ETL | 17 | 51 | 42 | - | - |
| Bandwidth (Hz/Px) | 285 | 1401 | 2122 | 810 | 670 |
| Matrix  Acquired  Interpolated | 288 x 288  576 x 576 | 102 x 102  204 x 204 | 84 x 124  168 x 248 | 128 x 128  256 x 256 | 367 x 416  734 x 832 |
| R-factor | 4 | 2 | 2 | 4 | 4 |
| b-values | - | Acquired 0 and 800, calculated 1400 | Acquired 0 and 800 | - | - |
| Time resolution (sec) | - | - | - | 11.60 | - |
| Acquisition time (min) | 2:33 | 3:33 | 2:06 | 5:15 | 6:04 |

mpMRI = multiparametric magnetic resonance imaging. DL= deep learning. TWIST = time-resolved angiography with interleaved stochastic trajectories. VIBE = volume-interpolated breath-hold examination. DWI = diffusion-weighted imaging. DCE-MRI = dynamic contrast-enhanced magnetic resonance imaging. Gd = gadolinium. SE = spin echo. FOV = field of view. EPI = echo-planar imaging. STIR = short tau inversion recovery. GE = gradient echo. Sag = sagittal. Cor = coronal. T = tesla. Tra = transversal. TE = echo time. TR = repetition time. TI = inversion time. NEX = number of excitations. ETL = echo train length. R-factor: reduction factor with parallel imaging acceleration.

**Supplementary Table 4.** Fluorodeoxyglucose positron emission tomography with computed tomography (FDG-PET/CT) protocol for the assessment of primary penile carcinoma (Discovery MI, GE Healthcare, Waukesha, WI, USA).

| **PET/CT parameters** | **PET** | **Low dose CT** |
| --- | --- | --- |
|  |  |  |
| FOV (mm) | 700 | 500 |
| Matrix | 384 | 512 |
| In-plane resolution (mm x mm)  Slice thickness (mm) | 1.8 x 1.8  2,79 | 0.97 x 0.97  0,625 |
| Scan parameters | 150 seconds / bed position (150 mm 25% overlap)  Four-ring digital LightBurst PET Detector with 198 mm axial coverage | Helical scan, rotational time: 1.0, detector coverage: 40 mm, thickness: 2.75 mm, pitch: 1.375, interval: 2.79 mm, SFOV: Large Body, kV: 120, Smart mA, min/max mA: 20/110, Noise index: 34. |
| Reconstruction parameters | VPFX, Q.Clear,  Beta factor: 400 | Thickness: 0.625 mm, Interval: 0.625 mm, DFOV: 500 mm, Recon Type: Standard, MAR, ASiR-V: AR 50 |
|  |  |  |

**ASiR** = adaptive statistical iterative reconstruction that uses statistical noise modeling to clean up noisy CT images. It begins with images generated by traditional Filtered Back Projection (FBP). It then iteratively refines the image, modeling and subtracting the statistical noise present in the raw data. **Beta factor** = a crucial penalty parameter in Bayesian penalized likelihood reconstruction (BSREM), controlling the trade-off between image noise reduction and lesion quantification accuracy; lower β values reduce noise but can overestimate activity, while higher β values improve quantitative accuracy but increase noise, with optimal values typically ranging from 300-500 for general use (e.g., 400 for PET/CT). **CT** = computed tomography is an imaging technique using X-rays to create cross-sectional images of the body. **DFOV** = (Display Field of View) reconstruction in CT determines the designated region *within* the SFOV that is mathematically reconstructed and displayed as the image. **FOV** = field of view. **kV** (kilovoltage) refers to the peak voltage applied to the X-ray tube. **LightBurst PET detector** = a digital positron emission tomography (PET) detector developed by GE HealthCare. It combines a small lutetium-based scintillator crystal array 3.95 mm (transaxial) × 5.3 mm (axial) × 25 mm (depth) with a Silicon Photomultiplier (SiPM) that provides high sensitivity and enables faster scans and lower radiation doses compared to older analog systems. **mA** = milliamperes refers to the X-ray tube current, controlling the number of X-ray photons produced, directly impacting image quality and patient dose (less noise with more mA/photons). **MAR** = metal artifact reduction. **Noise index** = a user-selected parameter that defines the target amount of image noise in the final reconstructed image. **PET** = positron emission tomography is a nuclear medicine imaging method to show the distribution of a small amount of radioactive tracer in the body. **Pitch** = a parameter that defines the relationship between the patient table movement and the width of the X-ray beam. It directly affects the speed of the scan, the radiation dose to the patient, and the image quality (spatial resolution and image noise). **Smart mA** = automatic exposure control to adjust X-ray tube current dynamically for optimal quality and dose. **Q.Clear** = proprietary Bayesian penalized-likelihood (BPL) image reconstruction algorithm developed by GE HealthCare. **SFOV** = (Small Field-of-View) the area directly covered by the detector, providing high-quality, accurate data within its bounds but cutting off anatomy extending beyond it. **VPFX** = VUE point FX reconstruction is a GE HealthCare 3D iterative algorithm for PET/CT imaging, combining Time-of-Flight (TOF) and Point Spread Function (PSF) modeling for better contrast, activity delineation, and quantitative accuracy.

**Supplementary Table 5.** Interpretation of correlation coefficients (ρ), κ values and effect sizes (r) [6-8].

| **Correlation coefficient (ρ)**  1.00 – 0.90  0.89 – 0.70  0.69 – 0.40  0.39 – 0.10  < 0.10 | **Interpretation**  Very strong correlation  Strong correlation  Moderate correlation  Weak correlation  Negligible correlation |
| --- | --- |
| **κ-value**  1.00 – 0.81  0.80 – 0.61  0.60 – 0.41  0.40 – 0.21  < 0.20 | **Interpretation**  Very good agreement  Good agreement  Moderate agreement  Fair agreement  Poor agreement |
| **Effect size (r)**  0.50  0.30  0.10 | **Interpretation**  Large effect  Medium effect  Small effect |

**Supplementary Table 6.** Human papillomavirus (HPV) genotypes in 41 HPV-positive patients with primary penile cancer.

| **Subtype** | **Patients (n=41)** |
| --- | --- |
| 16 | 28 (68) |
| 18 | 3 (7) |
| 31 | 5 (12) |
| 33 | 1 (2) |
| 45 | 2 (5) |
| 56 | 1 (2) |
| 42 | 1 (2) |
| 61 | 3 (7) |
| 54 | 2 (5) |
| 43 | 2 (5) |
| 66 | 1 (2) |
| 6 | 2 (5) |
| 39 | 2 (5) |
| 82 | 2 (5) |
| 52 | 1 (2) |

All results are number (percentage) of patients. Coinfection with multiple HPV types was common.

**Supplementary Table 7**. Association between the penile skip lesions and findings on multiparametric magnetic resonance imaging (mpMRI), fluorodeoxyglucose positron emission tomography with computed tomography (FDG-PET/CT) and histopathology.

| **Variable** | **p** |
| --- | --- |
| ADC from mpMRI (µmm^2^/s) | 0.203^7^ |
| SUV from FDG-PET/CT^1^ | 0.221^7^ |
| Histology  Largest tumor diameter^2^  Thickness/infiltration depth^3^  Involvement of urethra^4^  Involvement of corpus spongiosum  Involvement of corpus cavernosum^5^  T stage^5^  N stage^6^  Presence of lymph node metastases^6^ | 0.622^8^  0.487^8^  0.957^9^  0.179^9^  0.004^9^  0.014^9^  0.013^9^  0.025^9^ |
| HPV status | 0.382^9^ |

^1^FDG-PET/CT data not available (n=1). ^2^Missing histopathological data (n=9). ^3^Missing histopathological data (n=8). ^4^Missing histopathological data (n=2). ^5^T3 stage was corrected on the presence of skip lesions and defined as an infiltration of the tunica albuginea/corpus cavernosum by the primary tumor only. ^6^Missing histopathological data (n=9). ^7^p value from the independent samples t test. ^8^p value from the Mann-Whitney U test. ^9^p value from the Pearson chi-square test. ADC = apparent diffusion coefficient. FDG-PET/CT = fluorodeoxyglucose positron emission tomography with computed tomography. HPV = human papillomavirus. mpMRI = multiparametric magnetic resonance imaging. SUV = standardized uptake value.

**Supplementary Table 8.** Association between findings on multiparametric magnetic resonance imaging (mpMRI), physical examination, fluorodeoxyglucose positron emission tomography with computed tomography (FDG-PET/CT) and histopathology. Inconclusive results excluded from the analyses.

| **MpMRI** | **Histopathology** | | | | | | | **κ** | **Sensitivity**  **(95% CI)** | **Specificity**  **(95% CI)** | **PPV**  **(95% CI)** | **NPV**  **(95% CI)** |
| --- | --- | --- | --- | --- | --- | --- | --- | --- | --- | --- | --- | --- |
| Involvement of corpus spongiosum  Yes  No | Yes  43 (61)  2 (3) | | | | No  6 (8)  19 (27) | | | 0.74 | 96 (84-99) | 76 (55-89) | 88 (75-94) | 90 (68-98) |
| Involvement of urethra^1^  Yes  No | Yes  19 (27)  5 (7) | | | | No  3 (4)  41 (58) | | | 0.74 | 79 (58-91) | 93 (81-98) | 86 (65-96) | 89 (76-95) |
| Involvement of corpus cavernosum  Yes  No | Yes  10 (14)  0 (0) | | | | No  4 (6)  57 (80) | | | 0.78 | 100 (69-100) | 93 (84-98) | 71 (43-89) | 100 (94-100) |
| T stage  ≤ T1  T2  T3 | ≤ T1  20 (28)  5 (7)  0 (0) | | T2  2 (3)  29 (41)  4 (6) | | | T3  0 (0)  0 (0)  10 (14) | | 0.84^4^ | 80 (60-92)  83 (66-92)  100 (69-100) | 96 (84-99)  86 (70-94)  93 (83-98) | 91 (69-98)  85 (69-94)  71 (43-89) | 90 (77-96)  83 (67-92)  100 (94-100) |
| Lymph node metastases^2^  Yes  No | Yes  16 (23)  6 (8) | | | | No  4 (6)  31 (44) | | | 0.62 | 73 (51-87) | 89 (73-96) | 80 (57-92) | 84 (68-93) |
| N stage^2^  N0  N1  N2  N3 | N0  31 (44)  3 (4)  1 (1)  0 (0) | N1  5 (7)  6 (8)  0 (0)  1 (1) | | | N2  0 (0)  1 (1)  2 (3)  0 (0) | | N3  1 (1)  2 (3)  2 (3)  2 (3) | 0.65^4^ | 86 (70-94)  55 (26-80)  67 (15-96)  29 (7-68) | 71 (49-86)  87 (73-94)  94 (84-98)  98 (87-100) | 84 (68-93)  50 (24-76)  40 (10-81)  67 (15-96) | 75 (52-89)  89 (76-95)  98 (87-100)  91 (79-96) |
| **Physical examination** | **Histopathology** | | | | | | | **κ** | **Sensitivity**  **(95% CI)** | **Specificity**  **(95% CI)** | **PPV**  **(95% CI)** | **NPV**  **(95% CI)** |
| T stage  ≤ T1  T2  T3 | ≤ T1  15 (21)  10 (14)  1 (1) | | T2  5 (7)  19 (27)  11 (15) | | | T3  0 (0)  3 (4)  7 (10) | | 0.56^4^ | 58 (38-75)  54 (38-70)  78 (41-95) | 89 (76-95)  64 (47-78)  80 (68-89) | 75 (52-89)  59 (42-75)  37 (18-60) | 78 (65-88)  59 (43-73)  94 (83-98) |
| **FDG-PET/CT** | **Histopathology** | | | | | | | **κ** | **Sensitivity**  **(95% CI)** | **Specificity**  **(95% CI)** | **PPV**  **(95% CI)** | **NPV**  **(95% CI)** |
| Lymph node metastases^2, 3^  Yes  No | Yes  18 (25)  5 (7) | | | No  12 (17)  21 (30) | | | | 0.40 | 78 (57-91) | 64 (46-78) | 60 (42-76) | 81 (61-92) |
| N stage^2, 3^  N0  N1  N2  N3 | N0  21 (30)  11 (15)  1 (1)  0 (0) | N1  4 (6)  5 (7)  3 (4)  1 (1) | | | N2  0 (0)  2 (3)  1 (1)  0 (0) | | N3  1 (1)  4 (6)  1 (1)  1 (1) | 0.43^4^ | 62 (44-77)  42 (18-70)  33 (4-85)  14 (2-59) | 77 (55-90)  61 (46-75)  91 (79-96)  98 (86-100) | 81 (61-92)  23 (10-38)  17 (2-64)  50 (6-94) | 57 (38-73)  79 (62-90)  96 (85-99)  89 (77-95) |

^1^Missing histopathological data (n=2). ^2^Missing histopathological data (n=9). ^3^FDG-PET/CT data not available (n=1). ^4^Quadratic-weighted κ was estimated for ordinal variables; all other κ values are unweighted. Categorical variables are described by the number (percentage) of patients. CI = confidence interval. FDG-PET/CT = fluorodeoxyglucose positron emission tomography with computed tomography. κ = kappa**.** mpMRI = multiparametric magnetic resonance imaging. NPV = negative predictive value. PPV = positive predictive value.

**Supplementary Table 9.** Association between findings on multiparametric magnetic resonance imaging (mpMRI), physical examination, fluorodeoxyglucose positron emission tomography with computed tomography (FDG-PET/CT) and histopathology. Inconclusive results grouped with negative findings.

| **MpMRI** | **Histopathology** | | | | | | | **κ** | **Sensitivity**  **(95% CI)** | **Specificity**  **(95% CI)** | **PPV**  **(95% CI)** | **NPV**  **(95% CI)** |
| --- | --- | --- | --- | --- | --- | --- | --- | --- | --- | --- | --- | --- |
| Involvement of corpus spongiosum  Yes  No | Yes  43 (61)  2 (3) | | | | No  6 (8)  20 (28) | | | 0.75 | 96 (84-99) | 77 (57-89) | 88 (75-94) | 91 (69-98) |
| Involvement of urethra^1^  Yes  No | Yes  19 (27)  5 (7) | | | | No  3 (4)  42 (59) | | | 0.74 | 79 (58-91) | 93 (81-98) | 86 (65-96) | 89 (77-96) |
| Involvement of corpus cavernosum  Yes  No | Yes  10 (14)  0 (0) | | | | No  4 (6)  57 (80) | | | 0.78 | 100 (69-100) | 93 (84-98) | 71 (43-89) | 100 (94-100) |
| T stage  ≤ T1  T2  T3 | ≤ T1  21 (30)  5 (7)  0 (0) | | T2  2 (3)  29 (41)  4 (6) | | | T3  0 (0)  0 (0)  10 (14) | | 0.84^4^ | 81 (61-92)  83 (66-92)  100 (69-100) | 96 (84-99)  86 (70-94)  93 (84-98) | 91 (71-98)  85 (69-94)  71 (43-89) | 90 (77-96)  84 (68-93)  100 (94-100) |
| Lymph node metastases^2^  Yes  No | Yes  16 (23)  8 (11) | | | | No  4 (6)  34 (48) | | | 0.58 | 67 (46-83) | 89 (74-96) | 80 (57-92) | 81 (66-90) |
| N stage^2^  N0  N1  N2  N3 | N0  34 (48)  3 (4)  1 (1)  0 (0) | N1  7 (10)  6 (8)  0 (0)  1 (1) | | | N2  0 (0)  1 (1)  2 (3)  0 (0) | | N3  1 (1)  2 (3)  2 (3)  2 (3) | 0.65^4^ | 87 (72-95)  46 (22-72)  67 (15-96)  29 (7-68) | 65 (44-82)  88 (75-94)  95 (85-98)  98 (87-100) | 81 (66-90)  50 (24-76)  40 (9-81)  67 (15-96) | 75 (52-89)  86 (73-93)  98 (88-100)  92 (81-96) |
| **Physical examination** | **Histopathology** | | | | | | | **κ** | **Sensitivity**  **(95% CI)** | **Specificity**  **(95% CI)** | **PPV**  **(95% CI)** | **NPV**  **(95% CI)** |
| T stage  ≤ T1  T2  T3 | ≤ T1  15 (21)  10 (14)  1 (1) | | T2  5 (7)  19 (27)  11 (15) | | | T3  0 (0)  3 (4)  7 (10) | | 0.56^4^ | 58 (38-75)  54 (38-70)  78 (41-95) | 89 (76-95)  64 (47-78)  80 (68-89) | 75 (52-89)  59 (42-75)  37 (18-60) | 78 (65-88)  59 (43-73)  94 (83-98) |
| **FDG-PET/CT** | **Histopathology** | | | | | | | **κ** | **Sensitivity**  **(95% CI)** | **Specificity**  **(95% CI)** | **PPV**  **(95% CI)** | **NPV**  **(95% CI)** |
| Lymph node metastases^2, 3^  Yes  No | Yes  18 (25)  6 (8) | | | No  12 (17)  26 (37) | | | | 0.42 | 75 (54-88) | 68 (52-81) | 60 (42-76) | 81 (64-91) |
| N stage^2, 3^  N0  N1  N2  N3 | N0  26 (37)  11 (15)  1 (1)  0 (0) | N1  5 (7)  5 (7)  3 (4)  1 (1) | | | N2  0 (0)  2 (3)  1 (1)  0 (0) | | N3  1 (1)  4 (6)  1 (1)  1 (1) | 0.45^4^ | 67 (50-80)  38 (17-66)  33 (4-85)  14 (2-59) | 74 (52-88)  65 (51-77)  92 (81-96)  98 (88-100) | 81 (64-91)  23 (10-45)  17 (2-64)  50 (6-94) | 57 (38-73)  80 (64-90)  96 (86-99)  90 (79-96) |

^1^Missing histopathological data (n=2). ^2^Missing histopathological data (n=9). ^3^FDG-PET/CT data not available (n=1). ^4^Quadratic-weighted κ was estimated for ordinal variables; all other κ values are unweighted. Categorical variables are described by the number (percentage) of patients. CI = confidence interval. FDG-PET/CT = fluorodeoxyglucose positron emission tomography with computed tomography. κ = kappa**.** mpMRI = multiparametric magnetic resonance imaging. NPV = negative predictive value. PPV = positive predictive value.

**Supplementary Table 10.** Association between findings on multiparametric magnetic resonance imaging (mpMRI), physical examination, fluorodeoxyglucose positron emission tomography with computed tomography (FDG-PET/CT) and histopathology. Inconclusive results grouped with positive findings.

| **MpMRI** | **Histopathology** | | | | | | | **κ** | **Sensitivity**  **(95% CI)** | **Specificity**  **(95% CI)** | **PPV**  **(95% CI)** | **NPV**  **(95% CI)** |
| --- | --- | --- | --- | --- | --- | --- | --- | --- | --- | --- | --- | --- |
| Involvement of corpus spongiosum  Yes  No | Yes  43 (61)  2 (3) | | | | No  7 (10)  19 (27) | | | 0.72 | 96 (84-99) | 73 (53-87) | 86 (73-93) | 90 (68-98) |
| Involvement of urethra^1^  Yes  No | Yes  19 (27)  5 (7) | | | | No  4 (6)  41 (58) | | | 0.71 | 79 (58-91) | 91 (78-97) | 83 (61-93) | 89 (76-95) |
| Involvement of corpus cavernosum  Yes  No | Yes  10 (14)  0 (0) | | | | No  4 (6)  57 (80) | | | 0.78 | 100 (69-100) | 93 (84-98) | 71 (43-89) | 100 (94-100) |
| T stage  ≤ T1  T2  T3 | ≤ T1  20 (28)  6 (8)  0 (0) | | T2  2 (3)  29 (41)  4 (6) | | | T3  0 (0)  0 (0)  10 (14) | | 0.82^4^ | 77 (57-89)  83 (66-92)  100 (69-100) | 96 (84-99)  83 (67-92)  93 (84-98) | 91 (69-98)  83 (66-92)  71 (43-89) | 88 (75-94)  83 (67-92)  100 (94-100) |
| Lymph node metastases^2^  Yes  No | Yes  18 (25)  6 (8) | | | | No  7 (10)  31 (44) | | | 0.56 | 75 (54-89) | 82 (66-91) | 72 (51-86) | 84 (68-93) |
| N stage^2^  N0  N1  N2  N3 | N0  31 (44)  6 (8)  1 (1)  0 (0) | N1  5 (7)  8 (11)  0 (0)  1 (1) | | | N2  0 (0)  1 (1)  2 (3)  0 (0) | | N3  1 (1)  2 (3)  2 (3)  2 (3) | 0.63^4^ | 79 (64-89)  62 (34-83)  67 (15-96)  29 (7-68) | 74 (52-88)  82 (68-90)  95 (85-98)  98 (88-100) | 84 (68-93)  47 (25-70)  40 (10-81)  67 (15-96) | 68 (47-83)  89 (76-95)  98 (88-100)  92 (81-96) |
| **Physical examination** | **Histopathology** | | | | | | | **κ** | **Sensitivity**  **(95% CI)** | **Specificity**  **(95% CI)** | **PPV**  **(95% CI)** | **NPV**  **(95% CI)** |
| T stage  ≤ T1  T2  T3 | ≤ T1  15 (21)  10 (14)  1 (1) | | T2  5 (7)  19 (27)  11 (15) | | | T3  0 (0)  3 (4)  7 (10) | | 0.56^4^ | 58 (38-75)  54 (38-70)  78 (41-95) | 89 (76-95)  64 (47-78)  80 (68-89) | 75 (52-89)  59 (42-75)  37 (18-60) | 78 (65-88)  59 (43-73)  94 (83-98) |
| **FDG-PET/CT** | **Histopathology** | | | | | | | **κ** | **Sensitivity**  **(95% CI)** | **Specificity**  **(95% CI)** | **PPV**  **(95% CI)** | **NPV**  **(95% CI)** |
| Lymph node metastases^2, 3^  Yes  No | Yes  19 (27)  5 (7) | | | No  17 (24)  21 (30) | | | | 0.32 | 79 (58-91) | 55 (39-70) | 53 (35-69) | 81 (61-92) |
| N stage^2, 3^  N0  N1  N2  N3 | N0  21 (30)  16 (23)  1 (1)  0 (0) | N1  4 (6)  6 (8)  3 (4)  1 (1) | | | N2  0 (0)  2 (3)  1 (1)  0 (0) | | N3  1 (1)  4 (6)  1 (1)  1 (1) | 0.40^4^ | 54 (38-69)  46 (22-72)  33 (4-85)  14 (2-59) | 78 (57-91)  55 (41-69)  92 (81-96)  98 (88-100) | 81 (61-92)  21 (10-38)  17 (2-64)  50 (6-94) | 50 (34-66)  79 (62-90)  96 (86-99)  90 (79-96) |

^1^Missing histopathological data (n=2). ^2^Missing histopathological data (n=9). ^3^FDG-PET/CT data not available (n=1). ^4^Quadratic-weighted κ was estimated for ordinal variables; all other κ values are unweighted. Categorical variables are described by the number (percentage) of patients. CI = confidence interval. FDG-PET/CT = fluorodeoxyglucose positron emission tomography with computed tomography. κ = kappa**.** mpMRI = multiparametric magnetic resonance imaging. NPV = negative predictive value. PPV = positive predictive value.

**Supplementary Table 11.** Relationship between continuous variables and human papillomavirus (HPV) status.

| **Variables** | **HPV-positive** | **HPV-negative** | **Z** | **r** | **p** |
| --- | --- | --- | --- | --- | --- |
| Age (years) | 71 (57-77) | 74 (59-80) | -0.821 | -0.1 | 0.412^4^ |
| Largest diameter on mpMRI (mm) | 26 (18-37) | 31 (23-40) | -1.561 | -0.2 | 0.119^4^ |
| Thickness/infiltration depth on mpMRI (mm) | 10 (6-14) | 12 (8-15) | -1.203 | -0.1 | 0.229^4^ |
| Largest diameter on histopathology (mm)^1^ | 30 (20-40) | 28 (22-40) | -0.036 | < -0.1 | 0.972^4^ |
| Thickness/infiltration depth on histopathology (mm)^2^ | 9 (5-13) | 10 (4-18) | -0.774 | < -0.1 | 0.457^4^ |
| **Variables** | **HPV-positive** | **HPV-negative** | **t** | **r** | **p** |
| Imaging parameters of the tumor:  ADC from mpMRI (µmm^2^/s)  SUV from FDG-PET/CT^3^ | 819 (588-1100)  14.86 (10-20.4) | 985 (650-1164)  14.65 (9.4-22.4) | 4.311  -0.066 | 0.5  < -0.1 | <0.001^5^  0.948^5^ |

^1^Missing histopathological data (n=9). ^2^Missing histopathological data (n=8). ^3^FDG-PET/CT data not available (n=1). ^4^p value, Z score and an effect size (r) from the Mann-Whitney U test. ^5^p value, t value and an effect size (r) from the independent samples t test. Continuous variables are described by median (interquartile range). ADC = apparent diffusion coefficient. FDG-PET/CT = fluorodeoxyglucose positron emission tomography with computed tomography. HPV = human papillomavirus. mpMRI = multiparametric magnetic resonance imaging. SUV = standardized uptake value.

**Supplementary Table 12.** Association between findings on multiparametric magnetic resonance imaging (mpMRI), fluorodeoxyglucose positron emission tomography with computed tomography (FDG-PET/CT) and human papillomavirus (HPV) status. Inconclusive results included in the analyses.

| **MpMRI** | **HPV status** | | **p** |
| --- | --- | --- | --- |
| Involvement of corpus spongiosum  Yes  No  Inconclusive | Positive  28 (39)  13 (18)  1 (1) | Negative  21 (30)  8 (11)  0 (0) | 0.467 |
| Involvement of urethra  Yes  No  Inconclusive | Positive  10 (14)  28 (39)  0 (0) | Negative  12 (17)  20 (28)  1 (1) | 0.660 |
| Involvement of corpus cavernosum  Yes  No | Positive  7 (10)  34 (48) | Negative  7 (10)  23 (32) | 0.513 |
| T staging  ≤T1  T2  T3  Inconclusive | Positive  13 (18)  21 (30)  7 (10)  0 (0) | Negative  9 (13)  13 (18)  7 (10)  1 (1) | 0.582 |
| Lymph node metastases^1^  Yes  No  Inconclusive | Yes  12 (17)  21 (30)  1 (1) | No  8 (11)  16 (23)  4 (6) | 0.257 |
| N staging^1^  N0  N1  N2  N3  Inconclusive | Positive  21 (30)  6 (8)  5 (7)  1 (1)  1 (1) | Negative  16 (23)  6 (8)  0 (0)  2 (3)  4 (6) | 0.121 |
| DCE  Curve type 1  Curve type 2  Curve type 3  Cannot be assessed | Positive  0 (0)  7 (10)  32 (45)  2 (3) | Negative  1 (1)  7 (10)  21 (30)  1 (1) | 0.581 |
| **FDG-PET/CT** | **HPV status** | | **p** |
| Lymph node metastases^1,2^  Yes  No  Inconclusive | Positive  16 (23)  16 (23)  2 (3) | Negative  14 (20)  10 (14)  4 (6) | 0.445 |
| N staging^1,2^  N0  N1  N2  N3  Inconclusive | Positive  16 (23)  12 (17)  3 (4)  1 (1)  2 (3) | Negative  10 (14)  10 (14)  3 (4)  1 (1)  4 (6) | 0.797 |

^1^Excluded from the analysis (n=9). ^2^FDG-PET/CT data not available (n=1). FDG-PET/CT = fluorodeoxyglucose positron emission tomography with computed tomography. HPV = human papillomavirus. mpMRI = multiparametric magnetic resonance imaging. Categorical variables are described by the number (percentage) of patients.

**Supplementary Table 13.** Association between findings on physical examination and human papillomavirus (HPV) status.

| **Physical examination** | **HPV status** | | **p** |
| --- | --- | --- | --- |
| T staging  ≤T1  T2  T3 | Positive  13 (18)  18 (25)  10 (14) | Negative  7 (10)  14 (20)  9 (13) | 0.717 |

HPV = human papillomavirus. Categorical variables are described by the number (percentage) of patients.

**Supplementary Table 14.** Association between histopathology findings and human papillomavirus (HPV) status.

| **Histopathology** | **HPV status** | | **p** |
| --- | --- | --- | --- |
| Involvement of corpus spongiosum  Yes  No | Positive  26 (37)  15 (21) | Negative  19 (27)  11 (15) | 0.994 |
| Involvement of urethra^1^  Yes  No | Positive  14 (20)  25 (35) | Negative  10 (14)  20 (28) | 0.825 |
| Involvement of corpus cavernosum  Yes  No | Positive  5 (7)  36 (51) | Negative  5 (7)  25 (35) | 0.593 |
| T staging  ≤T1  T2  T3 | Positive  15 (21)  21 (30)  5 (7) | Negative  11 (15)  14 (20)  5 (7) | 0.853 |
| Lymph node metastases^2^  Yes  No | Positive  14 (20)  20 (28) | Negative  10 (14)  18 (25) | 0.660 |
| N staging^2^  N0  N1  N2  N3 | Positive  20 (28)  9 (13)  3 (4)  2 (3) | Negative  18 (25)  5 (7)  0 (0)  5 (7) | 0.172 |

^1^Missing histopathological data (n=2). ^2^Missing histopathological data (n=9). HPV = human papillomavirus. Categorical variables are described by the number (percentage) of patients.
